# Asked for It

**Published:** 1915-01

**Authors:** 


### Asked For It.

Raging and half mad with toothache, Biggs rushed to the dentist. Bearing untold agony, he tried to fix his attention upon the old magazines provided at the establishments of tooth luggers until, just on the last lap of endurance, he sank into the operating chair.

"Good heavens," exclaimed the dentist, as he surveyed the massive jaw of Biggs. "Had any advice about these teeth before?"

"Yes," gurgled Biggs. "Went to the chemist last night."

The dentist sniffed. "What idiotic thing did he tell you to do?" he demanded.

"To come to you!" murmured poor Biggs.—*New York Journal.*

